# *Gimap3* Regulates Tissue-Specific Mitochondrial DNA Segregation

**DOI:** 10.1371/journal.pgen.1001161

**Published:** 2010-10-14

**Authors:** Riikka Jokinen, Paula Marttinen, Helen Katarin Sandell, Tuula Manninen, Heli Teerenhovi, Timothy Wai, Daniella Teoli, J. C. Loredo-Osti, Eric A. Shoubridge, Brendan J. Battersby

**Affiliations:** 1 Research Program of Molecular Neurology and Institute of Biomedicine, Biomedicum Helsinki, University of Helsinki, Helsinki, Finland; 2 Montreal Neurological Institute and Department of Human Genetics, McGill University, Montreal, Quebec, Canada; 3 Department of Mathematics and Statistics, Memorial University, St. John's, Newfoundland, Canada; Max Planck Institute for Biology of Aging, Germany

## Abstract

Mitochondrial DNA (mtDNA) sequence variants segregate in mutation and tissue-specific manners, but the mechanisms remain unknown. The segregation pattern of pathogenic mtDNA mutations is a major determinant of the onset and severity of disease. Using a heteroplasmic mouse model, we demonstrate that Gimap3, an outer mitochondrial membrane GTPase, is a critical regulator of this process in leukocytes. Gimap3 is important for T cell development and survival, suggesting that leukocyte survival may be a key factor in the genetic regulation of mtDNA sequence variants and in modulating human mitochondrial diseases.

## Author Summary

Mitochondria are essential cellular organelles, which contain their own small circular genome in mammals. The mitochondrial genome is maternally inherited and encodes proteins critical for aerobic energy production. Mutations in mitochondrial DNA can lead to disruptions in aerobic energy production, which manifest as a wide spectrum of human clinical disorders. These mutations can segregate in mitotic and post-mitotic tissues, so that the proportion of the mutant genome can change with age, thereby affecting the clinical onset and severity of disease. The mechanisms regulating mtDNA segregation in mammals are not understood. We have used a mouse model of mtDNA segregation to elucidate the molecular basis of this process. Here, we demonstrate that the gene, Gimap3, can regulate mtDNA segregation in mouse white blood cells. This is the first gene identified to regulate mtDNA segregation in mammals, which is also an important factor in cell survival. Future work on this protein will provide critical insight into the pathways that regulate mitochondrial DNA in white blood cells, an important factor in the pathogenesis of human mitochondrial DNA mutations.

## Introduction

Mammalian mitochondrial DNA (mtDNA) is a maternally inherited high copy genome. Copy number ranges from 10^2^ to 10^5^ depending upon the cell type, and typically, there is a single haplotype or sequence variant in a cell (homoplasmy) [1]–[3]. Germline or somatic cell mutations in mtDNA lead to the co-occurrence of two or more sequence variants in a cell, a state known as heteroplasmy. In the absence of selection, the segregation of mtDNA sequence variants can be modeled as a random walk using two parameters: copy number and rate of turnover [4]. However, in some cases there is preferential selection for one mtDNA sequence variant over another, which depends upon the variant, tissue, and nuclear background.

Most human pathogenic mtDNA mutations are heteroplasmic, and typically oxidative phosphorylation function is impaired when the proportion of mutant mtDNA exceeds a critical threshold in the cell [5], [6], leading to a wide spectrum of clinical disorders, generally affecting tissues with a high aerobic demand [1]. Transmission of most mutations through the female germline is stochastic [7]; however, in somatic tissues, mtDNA mutations can have skewed segregation patterns depending upon the mutation, tissue, and pedigree [6]–[14]. For instance, there is negative selection for the A3243G mutation in tRNA^leu^ usually associated with MELAS (Mitochondrial Encephalomyopathy, Lactic Acidosis, Stroke-like episodes) in peripheral blood, but not in other tissues [15], [16]. However, this segregation pattern is not observed for other mitochondrial tRNA mutations, such as A8344G associated with MERRF (Myoclonic Epilepsy with Ragged-red fibers) [13], [17]. Thus, while both tRNA mutations impair mitochondrial translation, genetically these mutations are treated differently in the same cell types. To investigate the molecular basis for tissue-specific mtDNA segregation, we have used a heteroplasmic mouse model segregating two neutral mtDNA haplotypes derived from two old inbred mouse strains, BALB and NZB [18]. Transmission of these haplotypes through the female germline is neutral [18]; however, in post-natal life, the BALB mtDNA haplotype accumulates in hematopoietic tissues, while in the kidney and liver there is selection for the NZB haplotype [19]. In every other tissue investigated there is no preference for either mtDNA haplotype. The mechanisms for this mtDNA selection between tissues are apparently completely different [20], [21]. Previously, we demonstrated that nuclear-encoded genes regulate this selection process and mapped the quantitative trait loci (QTL) involved [22]. Further, we showed that selection for the BALB mtDNA haplotype in hematopoietic tissues can be completely eliminated in certain nuclear backgrounds [21]. In this study, we show that *Gimap3* is a critical gene for regulating mtDNA segregation hematopoietic tissues in this model.

## Results

**Figure 1:**
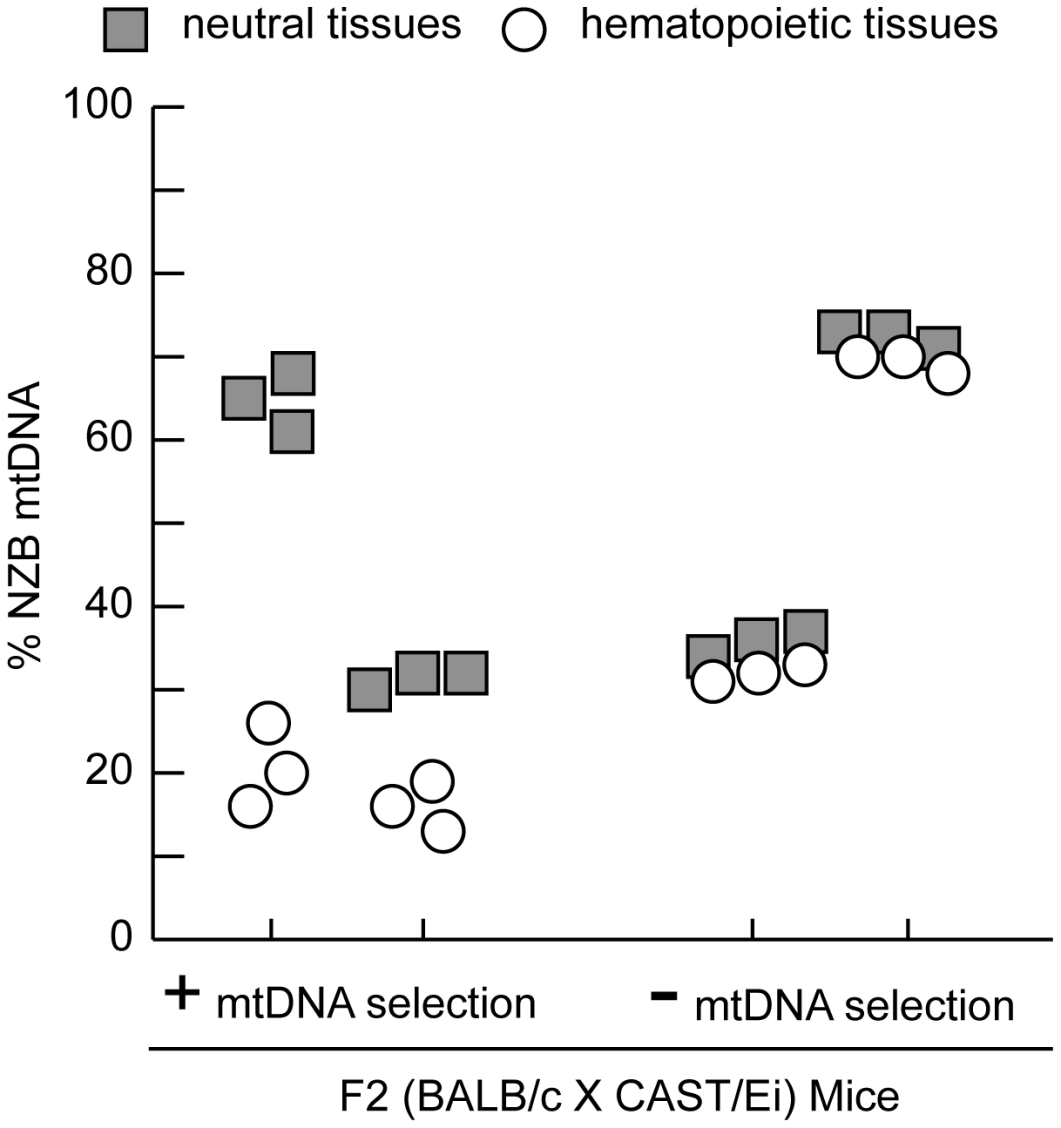
MtDNA segregation in hematopoietic tissues of 12-month-old heteroplasmic F2 (BALB/c X CAST/Ei) mice. A representative profile of mtDNA heteroplasmy levels in hematopoietic (spleen, peripheral blood, and bone marrow) and neutral tissues (heart, brain and skeletal muscle) from four 12-month-old F2 (BALB/c X CAST/Ei) mice illustrates the mtDNA segregation phenotypes. Mice were classified as having no (-) mtDNA selection if the % NZB mtDNA in hematopoietic tissues was similar to neutral tissues or having (+) mtDNA selection. Data is presented from mice with high (>60%) or moderate (35%) levels of NZB heteroplasmy in their neutral tissues.

Selection for the BALB mtDNA haplotype in hematopoietic tissues with age is rapid, proportional to the starting heteroplasmy level, and can be modeled as an exponential function [21]. The phenotype is robust, being found in a number of *Mus musculus domesticus* strains (DBA, 129Sv, NZB, C3H, C57BL/6J). In contrast, on the CAST/Ei mouse nuclear background, selection for the BALB mtDNA haplotype in hematopoietic tissues is completely abolished [21], suggesting that a combination of nuclear genes can completely regulate this process. To identify the genetic basis underlying this binary mtDNA segregation phenotype in hematopoietic tissues, we outcrossed heteroplasmic BALB/c females with CAST/Ei males to generate an F2 intercross (BALB/c X CAST/Ei). Mice were grouped into two phenotypes, based on either the absence or presence of mtDNA selection in the spleen (Figure 1). Mice were classified as having no mtDNA selection, if the % NZB mtDNA in hematopoietic tissues was similar to that of neutral tissues ie. those in which only random segregation is observed. All other mice were classified as positive for mtDNA selection, regardless of the rate of selection. In F2 mice, we found age-dependent regulation of this mtDNA segregation phenotype. At three months of age, approximately 40% of the F2 mice showed no mtDNA selection in the spleen, while at 12 months of age only 6% of F2 mice maintained the same phenotype. There was no difference between males and females.

**Figure 2:**
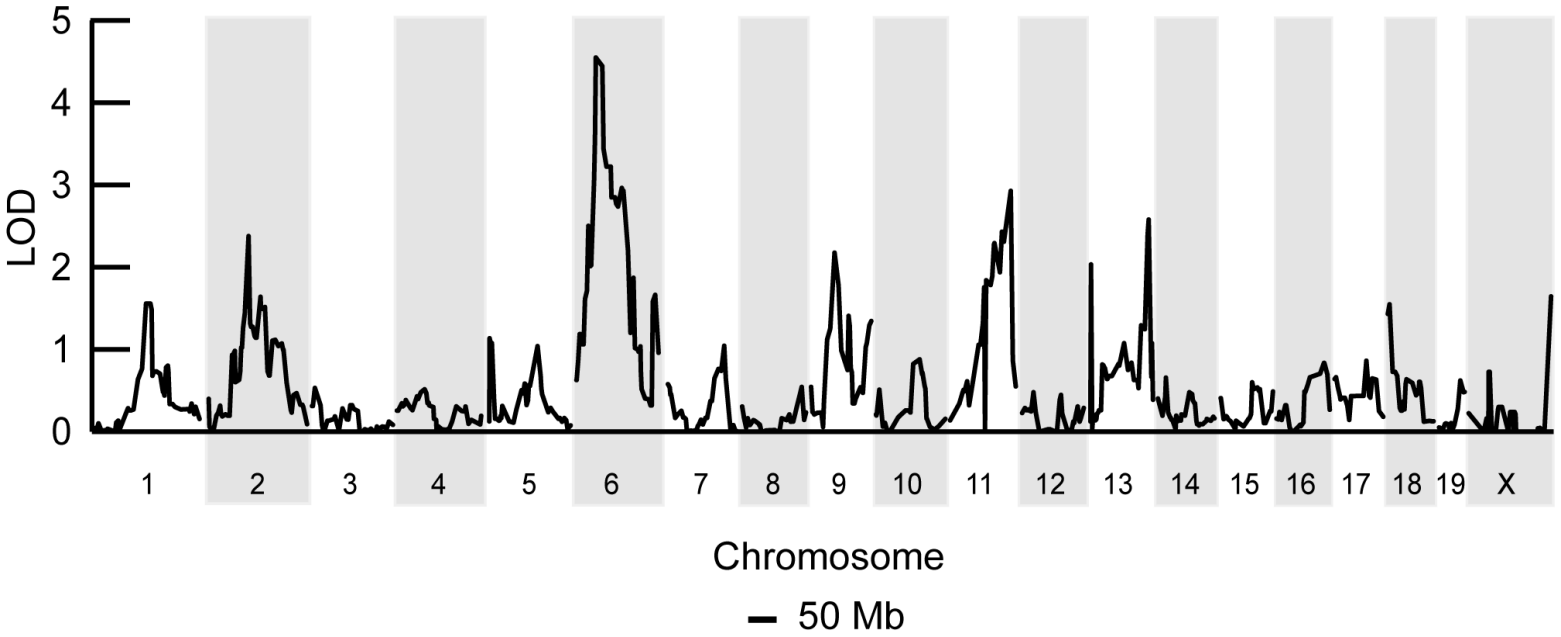
A chromosome 6 locus significantly affects mtDNA segregation in hematopoietic tissues. Genome-wide linkage analysis from 168 F2 (BALB/c X CAST/Ei) mice searching for loci regulating the loss of mtDNA selection. Only the chromosome 6 locus was significantly linked to this phenotype (LOD 4.6, genome-wide p = 0.007 with 10,000 permutations).

Clearly the genetic regulation of this binary mtDNA segregation phenotype is complex, yet we reasoned that at 12 months of age, two fully penetrant recessive loci could account for the frequency of such a phenotype. We performed a genome-wide linkage scan on 12 month old F2 mice (n = 168) using 680 SNPs to map loci regulating the absence of mtDNA segregation. We identified an 11 Mb interval on chromosome 6 (37.4–48.99 Mb) significantly linked to the loss of mtDNA selection (LOD 4.6, genome-wide p = 0.007 with 10, 000 permutations, Figure 2). No other loci across the genome reached statistically significant levels after the permutation analysis. However, we did detect two suggestive loci (p<0.63) [23], one on chromosome 11 (p = 0.310) and another on chromosome 13 (p = 0.557). We had previously mapped this same chromosome 6 locus as *Smdq- 3*, a QTL controlling the rate of mtDNA selection in the spleen at 12 months of age [22]. Together, these results confirms that chromosome 6 contains a gene(s) critical for the regulation of mtDNA segregation in hematopoietic tissues.

**Figure 3:**
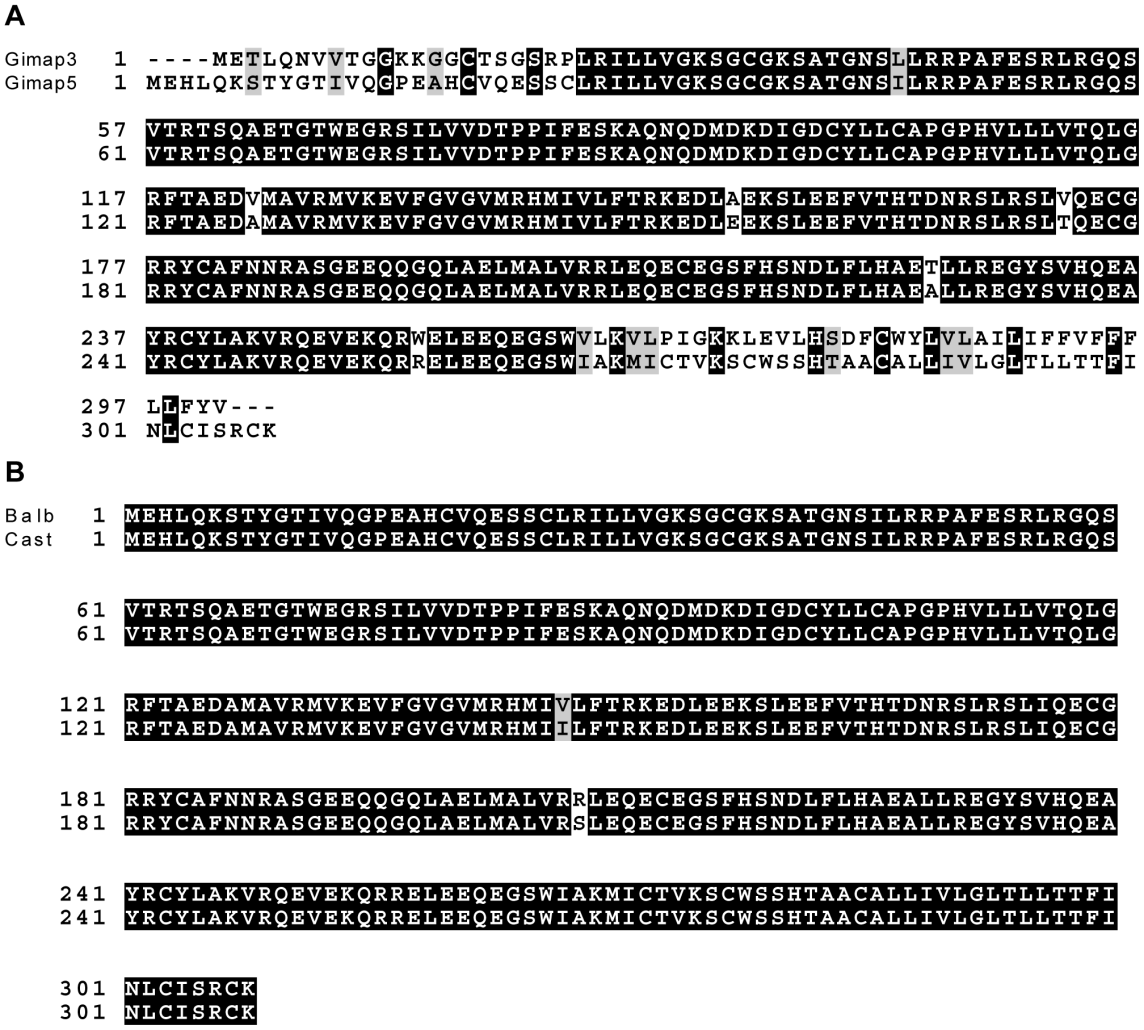
Gimap3 and Gimap5 protein sequences. A. ClustalW alignment of BALB Gimap3 and Gimap5 protein sequences. B. ClustalW alignment of BALB and CAST Gimap5.

To identify candidate genes within this 11 Mb interval, we searched for those annotated with a putative role in mitochondrial biology (GO:0005739 - mitochondrion) and found six matching this criterion (Table 1). Evaluating candidate genes for a tissue-specific role in mitochondrial biology is a difficult process, because most mitochondrial genes tend to be ubiquitously expressed. *Gstk1*, *Ndufb2* and *Mrps33* are ubiquitously expressed, and the latter two would presumably affect oxidative phosphorylation function. However, we and others have shown that there is no difference in respiratory chain function between NZB and BALB mtDNA haplotypes [20], [24]. Little is known of the putative kinase Adck2. In contrast, *Gimap3* and *Gimap5*, paralogues with 84% identity at the amino acid level (Figure 3A), have immune-related functions and make particularly attractive candidate genes because the mtDNA segregation phenotype occurs only in hematopoietic tissues.

**Table 1:** Chromosome 6 genes located between 37–49 Mb with a role in mitochondrial biology (GO:0005739).

Gene	Position (Mb)	Function
*Adck2*	39.52	kinase of unknown function
*Ndufb2*	39.54	Complex I subunit
*Mrps33*	39.75	mitochondrial ribosomal protein
*Gstk1*	42.19	protein disulfide oxidoreductase
*Gimap5*	48.66	immune related GTPase
*Gimap3*	48.71	immune related GTPase

**Figure 4:**
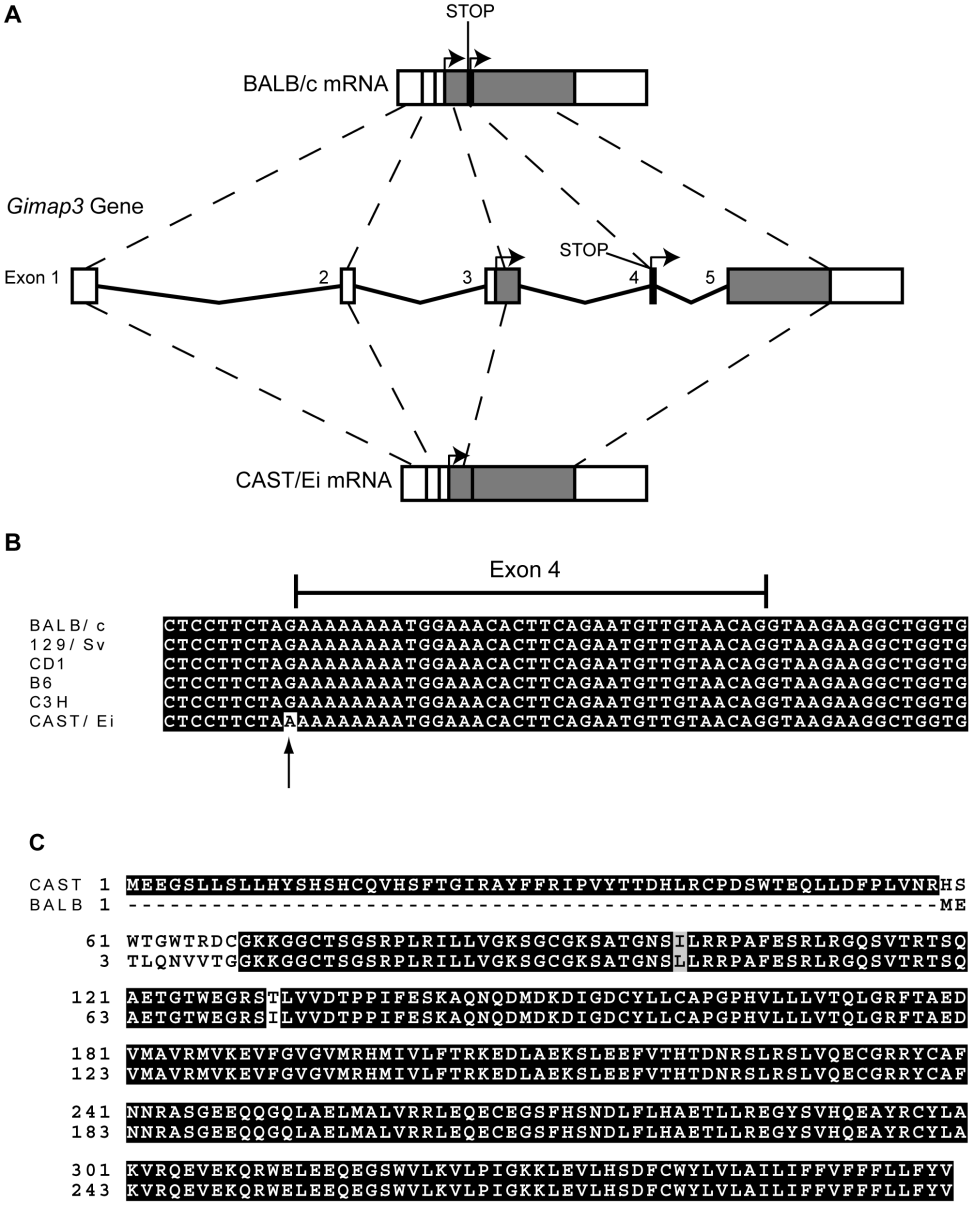
*Gimap3* gene structure and protein sequence in BALB/c and CAST/Ei mouse strains. A. Exon structure and splicing of *Gimap3*. An AUG start codon is present in both exon 3 and exon 4. In exon 4, upstream of AUG start codon is a stop codon. In the BALB/c allele all 5 exons are spliced together, so translation of the mature protein initiates at the second AUG start codon, with a predicted size of 34 kDa. In the CAST/Ei allele, a G-A transition in the splice acceptor site of exon 4 prevents its splicing into the mature mRNA, so exon 4 is missing and translation initiates from the first AUG start codon, predicting a protein of 41 kDa. B. Alignment of the *Gimap3* exon 4 and flanking intronic sequence from 5 *Mus musculus domesticus* strains, all of which are indistinguishable in the phenotype for mtDNA segregation compared to the *Mus musculus castaneus* CAST/Ei strain. C. ClustalW alignment of CAST and BALB Gimap3 protein sequences.

We sequenced the full-length cDNA of *Gimap3 and Gimap5* from total RNA extracted from BALB/c and CAST/Ei spleens. Gimap5 contained two missense changes (Val to Ile and Arg to Ser) between BALB and CAST variants (Figure 3B). Neither of these amino acid variants are evolutionarily conserved in other Gimap family members [25]. However, for *Gimap3* we found differential exon splicing with the CAST/Ei variant missing one of five exons. *Gimap3* consists of five exons with two in frame AUG start sites in exons 3 and 4. Exon 4 also contains a stop codon upstream of the second AUG start site, so when all five exons are spliced together, the second start is used for translation of the mature protein (Figure 4). In the CAST/Ei *Gimap3* mRNA, exon 4 is missing so translation starts from the first AUG (Figure 4), thereby altering the reading frame to produce a mature protein with an extra 58 amino acids at the N-terminus (Figure 4). We sequenced across exon 4 in genomic DNA from BALB/c and CAST/Ei and discovered a G to A transition in the splice acceptor site of exon 4 in CAST/Ei that prevents splicing of this exon into the mRNA (Figure 4). Since this mtDNA segregation phenotype is conserved among a variety of *Mus musculus domesticus* strains [22], we sequenced across exon 4 in four of these strains and found that the genomic sequence was identical to that of BALB/c (Figure 4). This altered mRNA splicing for the CAST/Ei allele changes considerably the Gimap3 protein sequence in the soluble domain of the protein, but does not affect the C-terminal transmembrane domain (Figure 4), which anchors and localizes it to the outer mitochondrial membrane [26].

**Figure 5:**
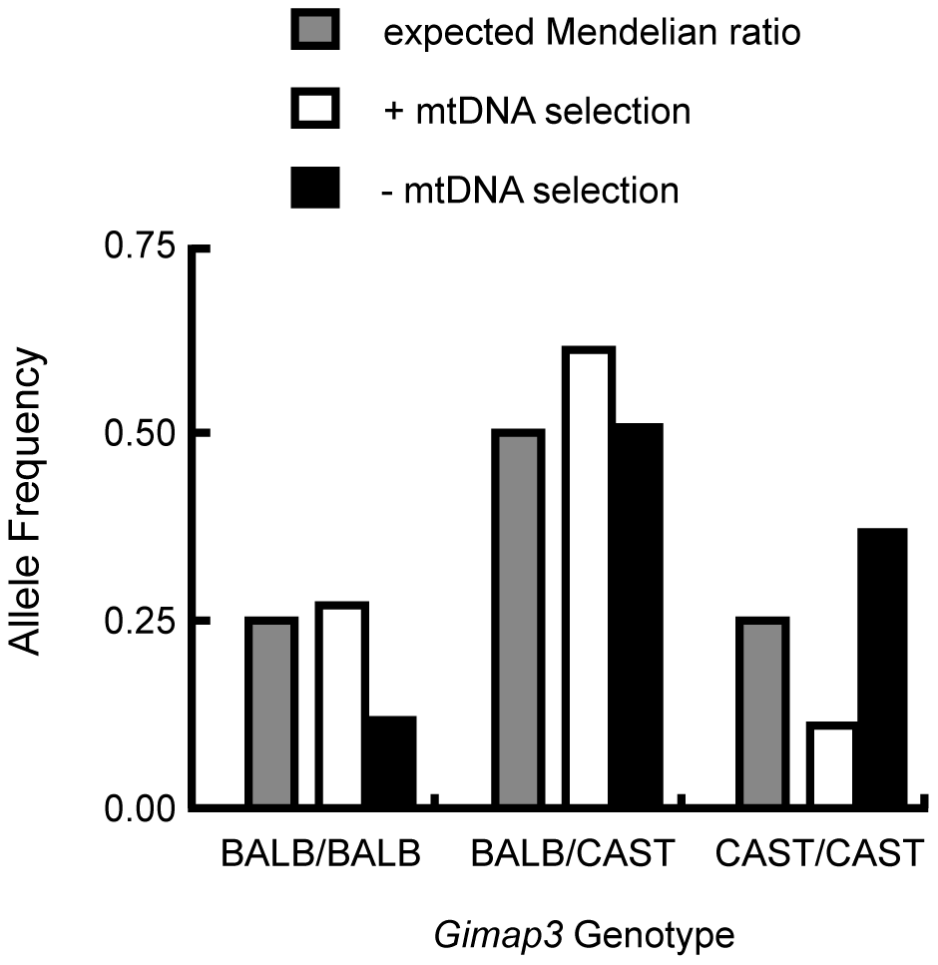
Enrichment of the CAST/Ei *Gimap3* allele in three-month-old F2 mice with no mtDNA selection. Three-month-old F2 (BALB/c X CAST/Ei) mice (n = 145) were classified into two groups, presence (+) (n = 88, 0.61) or absence (-) (n = 57, 0.39) of mtDNA selection, and then genotyped for their *Gimap3* alleles. Distributions were compared to the expected Mendelian ratios by Chi-square analysis (p = 0.00033).

MtDNA segregation in hematopoietic tissues is age-dependent, but it is unclear what role *Gimap3* has in younger mice. To test whether there was an association of the CAST/Ei allele with the loss of mtDNA selection in the spleen, we genotyped the *Gimap3* locus in three month old F2 mice (n = 145). Indeed, we observed a significant enrichment for the CAST/Ei allele in mice with no mtDNA selection and loss of the CAST/Ei allele in mice with mtDNA selection (Figure 5). This data suggests *Gimap3* plays an important role in mtDNA segregation in hematopoietic tissues independent of age and made *Gimap3* an attractive candidate gene.

**Figure 6:**
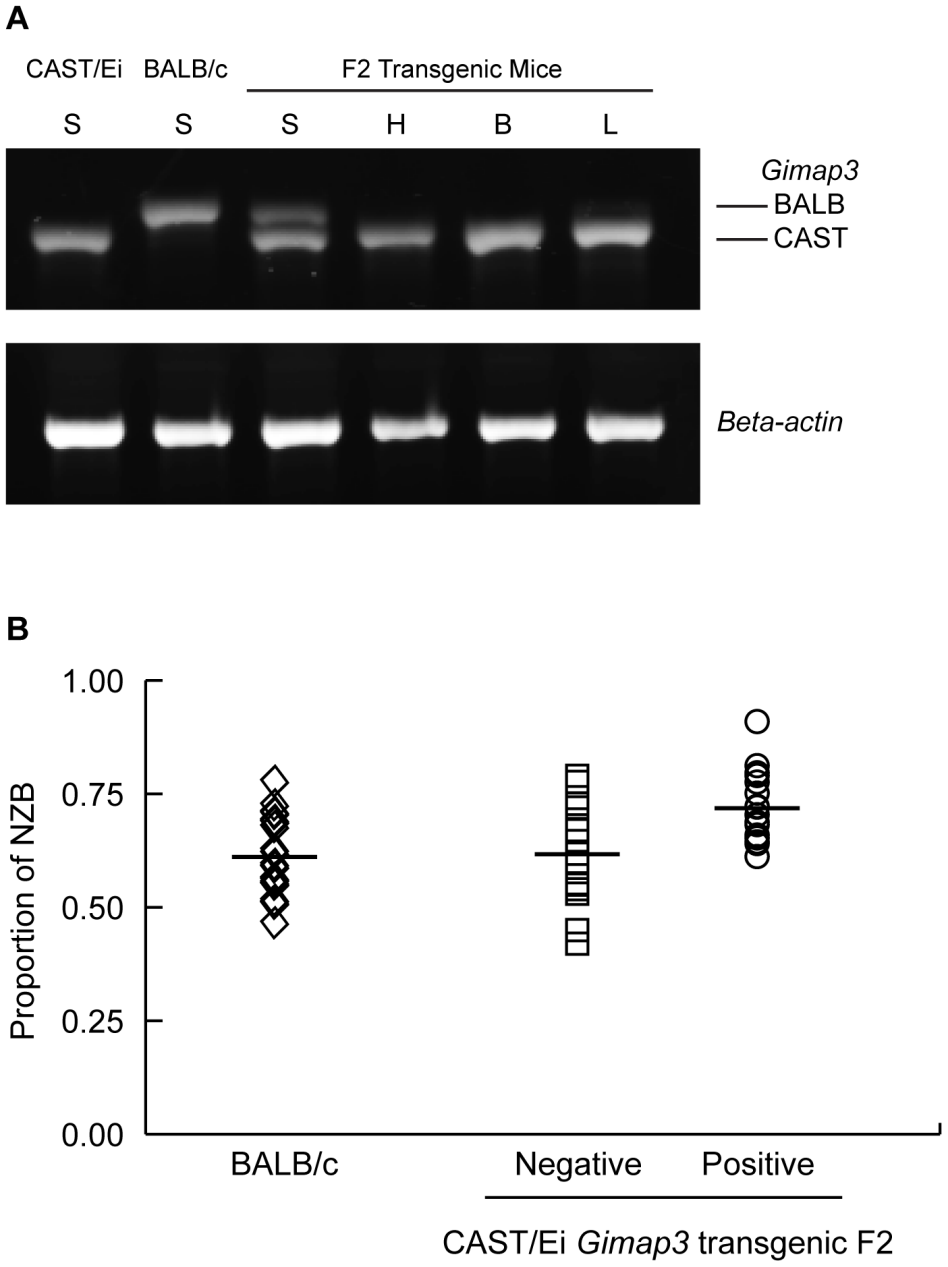
Transgenic expression of CAST/Ei *Gimap3* cDNA in heteroplasmic mice slows the rate of splenic mtDNA segregation. A. CAST/Ei *Gimap3* transgene expression across a number of mouse tissues (B-brain; L-lung; H-heart; S-spleen) driven off the ROSA26 promoter. Endogenous *Gimap3* expression from BALB/c or CAST/Ei spleen was loaded as a control. Equal amounts of total RNA were amplified by RT-PCR under the same conditions in each tissue. Beta-actin was used as a control. B. CAST/Ei *Gimap3* transgene expression in three-month-old mice significantly slows down the mean rate of mtDNA segregation in the spleen compared to littermate controls and the BALB/c heteroplasmic mouse model (ANOVA, p = 0.0011). Data are presented as a scatter plot with means indicated (bar). Transgene negative (n = 16) and positive (n = 17); BALB/c (n = 23).

To definitively test for a role of *Gimap3* in regulating mtDNA segregation, we generated transgenic mice overexpressing the Cast/Ei *Gimap3* cDNA driven off the ubiquitous ROSA26 promoter (Figure 6). The transgene was expressed ubiquitously, as expected from this promoter, and at a higher level than the endogenous *Gimap3* in the spleen (Figure 6). Transgenic males were crossed to heteroplasmic females and progeny sampled at three months of age. Our previous QTL mapping results demonstrated that the *Smdq-3* locus on chromosome 6 has an additive genetic effect, so our expectation was the CAST/Ei *Gimap 3* allele would slow the rate of mtDNA segregation in the spleen. Consistent with our expectation, overexpression of the CAST/Ei *Gimap3* in the spleen significantly slowed the rate of mtDNA segregation compared to littermate controls and our heteroplasmic mouse model on the BALB/c nuclear background (Figure 6). These results confirm *Gimap3* is an important regulator of hematopoietic mtDNA segregation.

**Figure 7:**
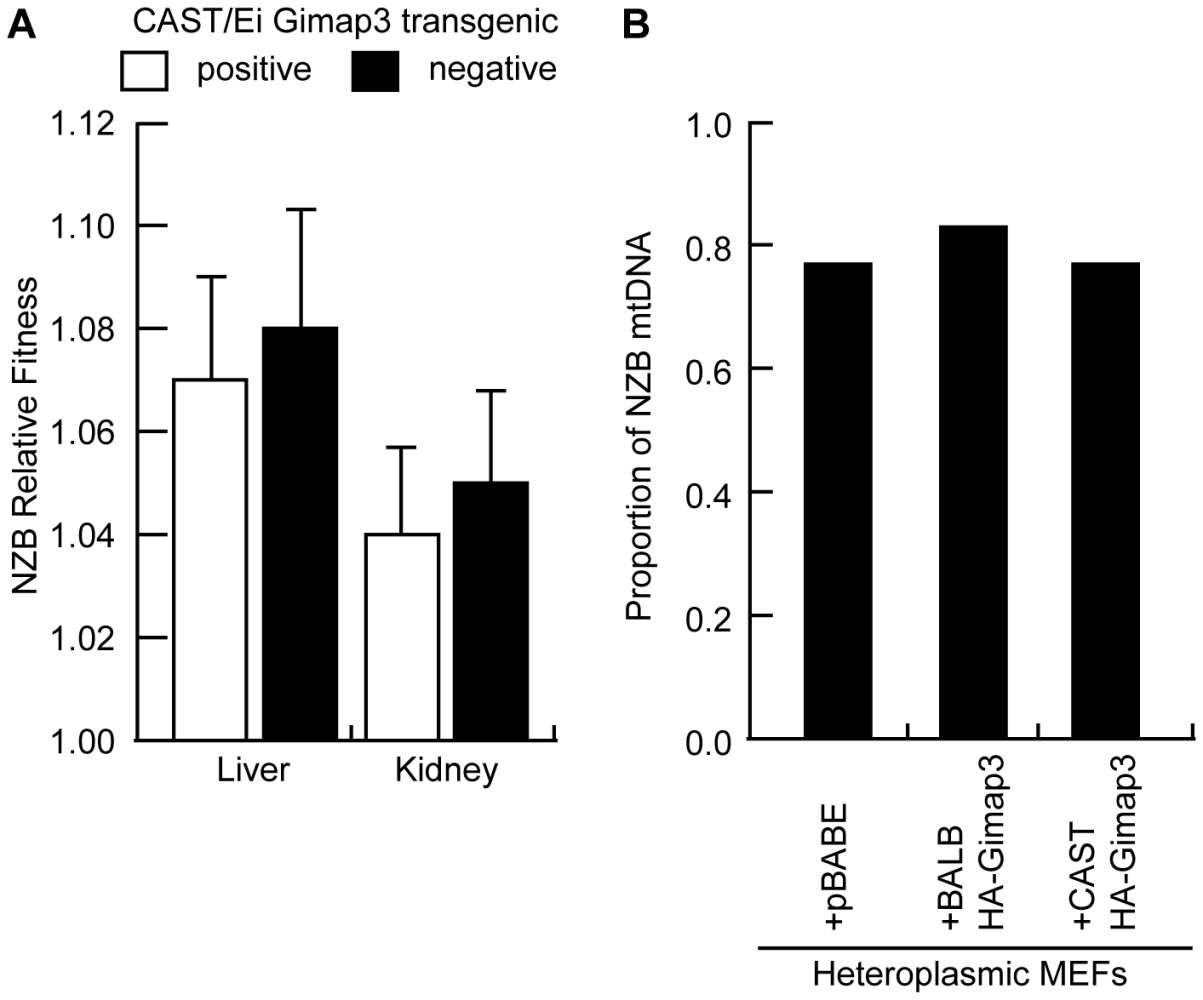
Ectopic expression of *Gimap3* has no effect on mtDNA segregation. A. Relative fitness of NZB mtDNA in the liver and kidney of F2 transgenic littermates positive or negative for the CAST/Ei *Gimap3* cDNA. Data are presented as means ± SD (transgene negative, n = 16; and positive, n = 17). B. Cultured heteroplasmic murine embryonic fibroblasts were transduced with BALB *Gimap3* or CAST *Gimap3* containing an N-terminal HA tag in pBABE, or with empty vector (pBABE). Cells were grown continuously in culture for 1 month. The change in NZB heteroplasmy in the bulk culture was determined comparing the level after 1 month of culture to the initial level before retroviral transduction.

Our results further support the hypothesis that the pathways regulating mtDNA segregation are indeed tissue or cell-specific. In our heteroplasmic mouse model, mtDNA selection for the NZB haplotype in the liver and kidney is regulated by different genes and with different kinetics [20], [22]. Ectopic expression of the CAST *Gimap3* transgene had no effect on NZB mtDNA selection in the liver or kidney (Figure 7), nor had any effect on mtDNA segregation in tissues which are neutral for selection in our mouse model, such as the brain, heart, lung, and skeletal muscle. Consistent with this finding, retroviral overexpression of the CAST Gimap3 in heteroplasmic murine embryonic fibroblasts had no effect on heteroplasmy levels (Figure 7). These results imply that a cell-specific context or pathway is also required to alter mtDNA segregation.

**Figure 8:**
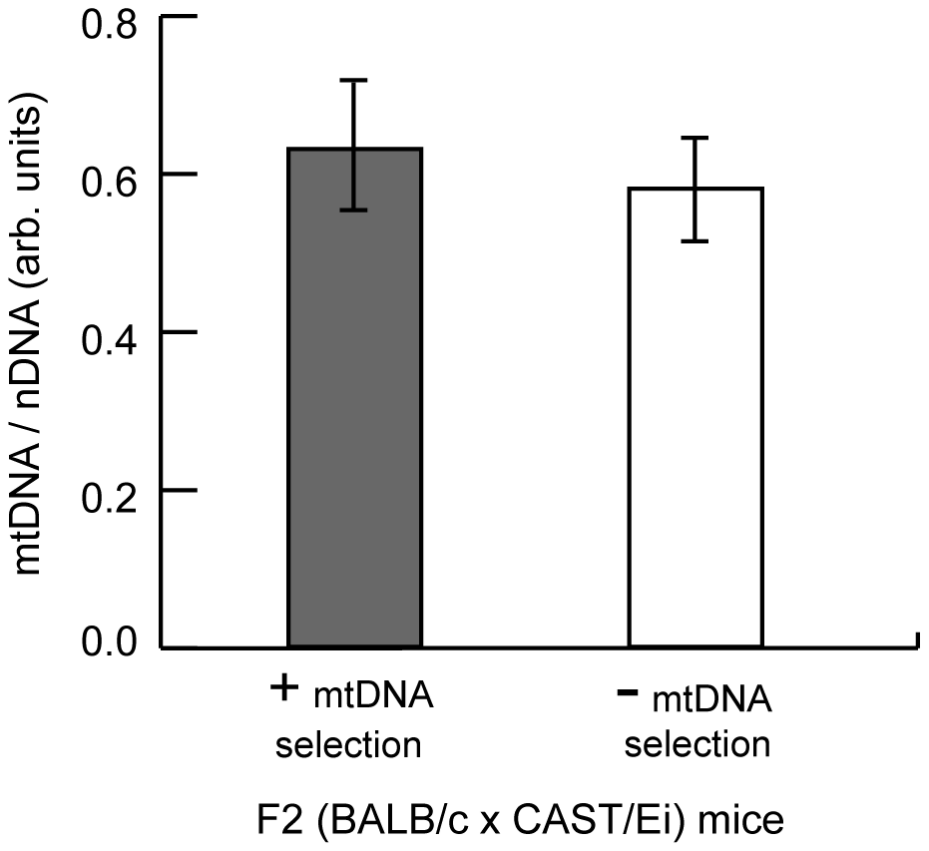
MtDNA copy number regulation in the spleen has no effect on mtDNA segregation. MtDNA copy number relative to nuclear DNA was measured in the spleen of three month F2 (BALB/c X CAST/Ei) mice with or without mtDNA selection. Data are presented as means ± SD (+ mtDNA selection, n = 32; - mtDNA selection, n = 13).

Mitochondrial genome copy number regulation has been proposed to influence the segregation of mtDNA haplotypes and human mtDNA mutations. To test whether changes in mtDNA copy number regulate mtDNA segregation in hematopoietic tissues, we measured the copy number in the spleen of F2 mice and found no difference between mice with either absence or presence of mtDNA selection (Figure 8). These data demonstrate that copy number regulation *per se* is not a major determinant for this particular mtDNA segregation phenotype, and that *Gimap3* expression has no role in regulating mtDNA copy number.

## Discussion

In this study, we identify the first nuclear-encoded gene that influences mtDNA segregation in mammals. Gimap3 is an outer mitochondrial membrane GTPase, which we show genetically can regulate the rate of mtDNA segregation in hematopoietic tissues. We also demonstrate that segregation of mtDNA haplotypes in mouse hematopoietic tissues is a complex genetic trait regulated with age but independent of mtDNA copy number. Variation in *Gimap3* alone does not account for the entire segregation phenotype for the following reasons. At three months of age, some mice homozygous for the CAST allele still exhibit mtDNA selection, the phenotype is age-dependent, and in our transgenic mice, overexpression of the CAST *Gimap3* had a quantitative effect on the rate of mtDNA segregation. These observations suggest other genes, such as the two suggestive loci detected on chromosome 11 and 13 in the linkage analysis are involved in the regulation of mtDNA segregation.

MtDNA selection in hematopoietic tissues of both humans and mice can be modeled as an exponential function, however, the rates are significantly different, up to 70 times faster in our mouse model than in humans [17]. In humans carrying the A3243G MELAS mutation, there is depletion of mtDNA independent of heteroplasmy level and age, which might be a secondary effect of the mutation and a driver for selection of wild type mtDNA [16]. However, the mechanism that leads to a decreased copy number and drives selection for the wild type mtDNA remains unknown. Rajasimha *et al.* [17] have postulated that selection against the A3243G MELAS mutation likely occurs in the stem cell population of rapidly dividing cells. Data from our mouse model do not support this mechanism of segregation, even though in our BALB/c heteroplasmic mouse model, selection for mtDNA occurs in leukocytes from both lymphoid and myeloid lineages [21]. In rapidly dividing colonic crypts there is no selection for mtDNA haplotypes [18], and, in our F2 (BALB/c X CAST/Ei) cross the frequency of mice that have lost mtDNA selection changes with age.

Gimap3 is a member of the conserved Gimap (GTPase of immunity-associated protein) gene cluster found only in vertebrates, with an orthologue in angiosperm plants [25]–[28]. Both Gimap3 and Gimap5 (a paralogue of Gimap3) contain a G1 to G5 switch GTPase, two coiled-coil motifs, and a hydrophobic conserved box [25]. In humans, only *GIMAP5* is a functional gene producing two splice variants with predicted molecular masses of 34.8 and 39.5 kDa, while *GIMAP3* appears to be a pseudogene [25]. Very little is known about protein function, and in particular the role of the GTPase domain and the conserved box remain an enigma. These two proteins are critical for T cell development and cell survival, and shown to interact with anti-apoptotic Bcl-2 family members, but the mechanisms are not understood [27]. Gimap5 was originally identified as the factor responsible for the severe T cell lymphopenia in the diabetes prone BioBreeding rat [29], although in mice loss of Gimap5 function produces a broader and more severe phenotype, which includes a leukocyte developmental defect, liver dysfunction, and lethality (median age of death around 14–15 weeks) [30]. The CAST/Ei variant of Gimap3 only differs at the N-terminus, leaving intact all of the known functional domains of the protein, including the C-terminus required for membrane insertion and localization. How these extra 58 amino acids in the CAST/Ei Gimap3 variant affect protein function requires further characterization of Gimap3 in leukocytes.

How can an outer mitochondrial membrane protein regulate mtDNA segregation in hematopoietic tissues? Selection for mtDNA haplotypes can only be directed at two levels, either at the DNA sequence itself or at the proteins encoded within it. Analysis of Gimap3 protein sequence does not support a direct physical interaction with mtDNA, because the protein does not appear to span both mitochondrial membranes into the matrix space in order to facilitate such an interaction. One possibility is that Gimap3 acts as a node or switch on the outer membrane for a retrograde signaling cascade involving mitochondrial peptide export, a process that occurs across eukaryotes [31]–[34]. Bacteria use peptide export-import as a control circuit to regulate processes, such as nutrient uptake and sporulation [35]. Further work on Gimap3 will establish its function within mtDNA segregation and whether peptide export or cell survival are involved.

## Methods

### Ethics Statement

These studies were approved by the McGill University Animal Care Committee and The Regional State Administrative Agency of Southern Finland (ESAVI).

### Mice and Breeding

To produce mice for the genome scan, female BALB/c mice heteroplasmic for the BALB and NZB mtDNA haplotypes were outcrossed to male CAST/Ei mice to generate an F1, which were then intercrossed to obtain F2 progeny. Transgenic mice were made by cloning the CAST/Ei cDNA of *Gimap3* into the EcoRI site of pBroad3 (Invivogen), which was then microinjected into fertilized FVB embryos. Founders were screened for the transgene, germline transmission, and autosomal inheritance. Transgenic mice were crossed to BALB/c, and the resulting F1 males crossed to heteroplasmic BALB/c females to generate heteroplasmic littermates.

### Phenotyping

Tissues were collected from mice at 3 and 12 months of age and DNA extracted by conventional methods. Heteroplasmy levels were determined across tissues and the mtDNA segregation phenotype in hematopoietic tissues done according to Battersby et al. [21]. Only animals with an initial level of NZB heteroplasmy above 20% were included in the analysis. Relative fitness values for NZB mtDNA in the kidney and liver were calculated as previously described [22].

### MtDNA Quantitation

Relative levels of mtDNA to nuclear DNA were determined using SYBR Green (Kapa Biosystems) on a Bio-Rad CFX96 thermal cycler with primers for mtDNA (forward 5′- GAGCATCTTATCCACGCTTCC, reverse 5′-GGTGGTACTCCCGCTGTAAA) and the single copy nuclear-encoded gene beta-2 microglobulin (forward 5-TGTCAGATATGTCCTTCAGCAAGG, reverse 5-TGCTTAACTCTGCAGGCGTATG). Samples and standards were run in triplicate and used only after comparing the post-run amplification efficiencies.

### Genotyping

The Illumina Medium Density Linkage Panel was used for SNP genotyping of mouse heart genomic DNA. From a total of 1449 markers on the panel, 680 SNPs were informative between BALB/c and CAST/Ei. The *Gimap3* allele was genotyped in genomic DNA by PCR using primers (forward 5′- ACGTGCACAGACCCATTTCT, reverse 5′- GTGCTGGAGGGAAGTTTGTC) and then digested Hpy188III and separated on agarose gels. Mice were screened for the presence of the transgene using a PCR assay that amplified the *Gimap3* CAST/Ei cDNA and the BALB/c gene (forward 5′-CATACCGTCACACCATCTGC, reverse 5′-CTTTTACCGCAGCCAGATTT), which amplifies a 320 bp fragment from the cDNA and a 1700 bp fragment from the gene.

### RNA Analysis

All tissues sampled were frozen in liquid nitrogen and stored at −80°C. Total RNA was extracted with Trizol (Invitrogen) then treated with DNaseI to eliminate potential DNA contamination. *Gimap3* cDNAs were amplified from BALB/c and CAST/Ei spleens by RT-PCR (Qiagen) with primers (forward 5′- TCCTGCCTGAGAGACTGTTG, reverse 5′- TGTGAGTGATCCCAATCCAC). Transgene and endogenous *Gimap3* expression was measured by RT-PCR using equal amounts of total RNA with primers for Gimap3 (forward 5′- TGGACTTCCCATTGGTAAACA, reverse 5′-ACCCCAAAGACCTCCTTCAC) and beta-actin (forward 5′ -TCACCCACACTGTGCCCATCTAC, reverse 5′ -GAGTACTTGCGCTCAGGAGGAGC).

### Retroviral Constructs

Full-length cDNAs were cloned into a Gateway (Invitrogen) converted pBABE-puro retroviral expression vector and transfected into the Phoenix amphotropic packaging line to transiently produce virus, which was then used to infect NIH3T3 or heteroplasmic murine embryonic fibroblasts.

### Statistical Analysis

Linkage analysis was carried out by regression at the markers under a logistic regression model and an allele dosage mode of inheritance. The genome wide corrected p-values were based on a 10,000 permutation sample. Allele distributions of *Gimap3* in three month old F2 mice were analyzed by Chi-Square analysis comparing to an expected Mendelian distribution. The effect of the CAST/Ei transgene on mtDNA segregation was analyzed in datasets first for normality, followed by ANOVA and posthoc testing.
